# Cross-Scale Spectral Calibration for Spatiotemporal Fusion of Remote Sensing Images

**DOI:** 10.3390/s26072090

**Published:** 2026-03-27

**Authors:** Yishuo Tian, Xiaorong Xue, Jingtong Yang, Wen Zhang, Bingyan Lu, Xin Zhao, Wancheng Wang

**Affiliations:** School of Electronics and Information Engineering, Liaoning University of Technology, Jinzhou 121001, China

**Keywords:** spatiotemporal fusion, cross-scale spectral inconsistency, spectral fidelity, spatial detail preservation, remote sensing, image fusion

## Abstract

Spatiotemporal fusion aims to generate remote sensing images with both high spatial and high temporal resolution by integrating multi-source observations. However, significant spectral inconsistencies often arise when fusing images acquired at different spatial scales, which severely degrade the radiometric fidelity and temporal reliability of the fused results. Most existing methods focus on enhancing spatial details or temporal consistency, while the cross-scale spectral discrepancy between coarse- and fine-resolution images has not been sufficiently addressed. To tackle this issue, we propose a cross-scale spectral calibration framework for spatiotemporal fusion (XSC-Net), which explicitly models and corrects spectral responses across different spatial scales. The proposed method introduces a spatial feature refinement block to enhance spatially discriminative structures and a hierarchical spectral refinement block to adaptively calibrate channel-wise spectral representations. By jointly exploiting spatial and spectral correlations, the proposed framework effectively suppresses spectral distortion while preserving fine spatial details. Extensive experiments on the public CIA and LGC datasets indicate that XSC-Net compares favorably with state-of-the-art methods, demonstrating superior performance over established baselines. Furthermore, ablation studies verify the efficacy and contribution of the proposed architectural components.

## 1. Introduction

Remote sensing imagery characterized by simultaneous high spatial and temporal resolution plays an indispensable role in Earth observation missions, particularly in domains such as precision agriculture (for example, crop growth monitoring) and environmental dynamic analysis [1]. However, constrained by physical sensor designs and transmission bandwidth limitations, acquiring such optimal data directly from a single satellite platform remains a significant challenge. For instance, while the MODIS sensor offers daily observations, it is limited by low spatial resolution (typically 250 m–1 km; hereafter referred to as “coarse images”). Conversely, the Landsat sensor captures fine-grained spatial details (typically 30 m; hereafter referred to as “fine images”), yet it is hampered by prolonged revisit cycles and susceptibility to cloud contamination [2,3,4,5]. To bridge this observational gap, Spatiotemporal Fusion (STF) technology has emerged. STF aims to synergize the complementary strengths of multi-source sensors to generate synthetic image sequences possessing both high spatial and temporal resolution (hereafter referred to as “fused images”), thereby facilitating the continuous monitoring of heterogeneous land surface dynamics.

Over the past few decades, spurred by diverse application contexts and varying a priori assumptions, a plethora of STF methodologies have emerged in the academic community [6,7]. These approaches can be broadly categorized into four distinct paradigms: Weight-function-based, Unmixing-based, Hybrid, and Learning-based methods. Typically, these algorithms necessitate the input of one or more pairs of coarse- and fine-resolution images—acquired at distinct timestamps—to serve as references for inferring and reconstructing the fused image on the prediction date. The first category, Weight-function-based methods, operates on the premise that neighboring pixels exhibiting spectral similarity in the fine image undergo similar temporal variations in the coarse image [8]. Based on this assumption, these algorithms employ a sliding window strategy to search for similar pixels, assigning weights determined by spectral discrepancy, temporal distance, and spatial distance to predict the value of the central pixel. A prominent example is the Spatial and Temporal Adaptive Reflectance Fusion Model (STARFM) and its variants [8,9]. Despite their extensive application, these methods remain sensitive to the selection of window size and often struggle to accurately capture abrupt, non-linear changes. Unmixing-based methods are predominantly grounded in the Linear Spectral Mixture Model (LSMM). These approaches leverage the rich spatial information provided by fine images to determine the granularity within coarse pixels, effectively ‘unmixing’ the coarse data to recover high-frequency spatial details [6]. For instance, the Multi-sensor Multi-resolution Technique (MMT) predicts fine pixels by unmixing coarse pixels based on linear spectral mixing theory [7], and is widely regarded as the pioneering approach introducing unmixing into spatiotemporal data fusion. While these methods demonstrate robustness in scenarios featuring significant categorical changes, their performance is highly contingent upon the accuracy of endmember classification or abundance estimation. Hybrid methods aim to synergize the strengths of the aforementioned strategies to simultaneously address gradual phenological variations and abrupt land cover transitions [10]. Among traditional spatiotemporal fusion algorithms, hybrid approaches have garnered extensive research attention due to their flexibility and superior performance.

The aforementioned three categories of methodologies are primarily grounded in physical assumptions or reconstruction-based rules and are collectively classified as traditional spatiotemporal fusion algorithms. Conversely, with the continuous advancement of machine learning and deep learning, learning-based data-driven approaches have garnered increasing attention within the domain of spatiotemporal fusion. In contrast to traditional paradigms, deep learning-based approaches possess the capability to automatically extract prior knowledge and model complex non-linear relationships via trained architectures. Leveraging the robust performance of Convolutional Neural Networks (CNNs) [11,12], a multitude of contemporary STF algorithms employ CNNs to learn non-linear mappings of remote sensing imagery, thereby enhancing algorithmic performance. Beyond standard CNN architectures, drawing inspiration from image super-resolution tasks, certain studies have incorporated Generative Adversarial Networks (GANs) into the training of STF models [13]. The distinction of GAN-driven approaches lies in their objective: rather than solely modeling precise pixel-wise relationships, they utilize adversarial training to reconstruct more realistic high-frequency texture details.

In summary, while deep learning-based approaches have achieved notable advancements, several critical challenges remain unaddressed. Primarily, simple feature concatenation is insufficient to fully exploit the complex interaction mechanisms between the temporal information of coarse images and the spatial information of fine images. More critically, in their pursuit of minimizing pixel-level errors, the majority of existing methodologies often compromise structural fidelity. In particular, the widely adopted Softmax-based attention mechanism is highly prone to inducing an Over-smoothing Effect in feature weights. This phenomenon causes the model to lose critical high-frequency details when leveraging fine imagery for guidance, resulting in texture blurring within heterogeneous regions (e.g., distinct agricultural ridges or minute man-made structures) in the final fused imagery. This issue is particularly acute in spatiotemporal fusion tasks. Since the reconstruction of high-resolution textures relies on cross-scale inference, high-frequency information is inherently fragile. Consequently, the smoothing effect induced by competitive attention mechanisms is significantly exacerbated in heterogeneous regions.

In this study, we focus on the problem of cross-scale spectral inconsistency in spatiotemporal fusion and propose a cross-scale spectral calibration framework to enhance radiometric fidelity and spatial consistency across multi-resolution observations. Many existing deep learning models, such as DCSTFN [12] or GAN-STFM [13], typically employ rudimentary channel concatenation or element-wise addition to fuse coarse and fine image features. This shallow interaction overlooks the semantic gap between features from heterogeneous sensors, making it difficult to effectively calibrate the spectral deviations between them. To overcome this limitation, XSC-Net introduces a Spectral Feature Rectification Block (SFRB). Unlike approaches that passively accept input features, the SFRB leverages the global contextual information of the coarse image to actively learn a rectification vector. This vector explicitly recalibrates the spectral distribution of the fine image, thereby ensuring spectral consistency in the fused image at the feature level. Furthermore, in terms of spatial texture restoration, traditional Softmax-based attention mechanisms enforce a constraint where the sum of weights across all positions must equal one. This ‘competitive’ normalization implies that when multiple significant features exist within a local region (e.g., dense agricultural ridges or complex building clusters), the weights of secondary features are forcibly suppressed, leading to detail loss. To mitigate this, we design a High-Frequency Structure Retention Block (HFRB), which innovatively employs a Sigmoid activation strategy as a substitute for Softmax. The Sigmoid function permits the attention map to exhibit a multi-hotspot response, ensuring that every edge and texture detail within the fine image can be independently activated and preserved, thus fundamentally circumventing the over-smoothing problem. Finally, traditional methods often adopt static weighting or globally unified rules when handling phenological changes; these strategies frequently fail to adapt to rapid local variations, resulting in “ghosting” artifacts. XSC-Net integrates a Dynamic Temporal Gating Mechanism (DTGM), which adaptively “gates” the pass-through rate of reference information by perceiving pixel-level temporal differences. This achieves an optimal balance between preserving spatial details and adapting to temporal dynamics. In summary, the main contributions of this work are as follows:

(1) To enhance spatial discrimination under scale mismatch, a spatial feature refinement mechanism is introduced to adaptively emphasize structurally informative regions while suppressing spatial distortion
(2) To correct spectral discrepancies across different resolutions, a hierarchical spectral refinement mechanism is developed to perform channel-wise spectral calibration, thereby improving spectral fidelity while preserving fine spatial details.
(3) To alleviate temporal inconsistencies during spatiotemporal fusion, a dynamic temporal gating mechanism is introduced to adaptively model pixel-wise temporal discrepancies, thereby suppressing ghosting artifacts and enhancing temporal reliability.

The remainder of this paper is organized as follows: Section 2 provides a comprehensive review of the related literature. Section 3 elaborates on the methodological details of the proposed framework. Section 4 presents the experimental settings and reports the comparative results. Finally, Section 5 draws the conclusions.

## 2. Related Work

Conventional spatiotemporal fusion algorithms are primarily grounded in physical models or reconstruction-based rules. As a pioneering algorithm in this domain [8], the Spatial and Temporal Adaptive Reflectance Fusion Model (STARFM) operates on the premise that the temporal spectral variations of coarse and fine imagery for identical land features are consistent. Specifically, this method predicts the central pixel value by aggregating weighted spectrally similar pixels within a sliding window. However, a notable limitation of STARFM is its suboptimal performance in heterogeneous landscapes. To mitigate this issue, the Enhanced STARFM (ESTARFM) introduced conversion coefficients to address the mixed pixel problem [9], thereby significantly improving prediction accuracy in scenarios involving complex land cover transitions. Despite these advancements, ESTARFM requires two pairs of reference images as input and suffers from high computational complexity, rendering it challenging to deploy over large-scale regions. Another representative methodology is the Flexible Spatiotemporal Data Fusion (FSDAF) model, which ingeniously integrates spectral unmixing with Thin Plate Spline (TPS) interpolation [10]. The primary advantage of FSDAF lies in its requirement of only a single reference image pair, alongside its capability to capture both gradual phenological changes and abrupt land cover events. Although these traditional methods have dominated the field for decades, they are fundamentally constrained by the linear spectral mixing assumption. When confronted with complex non-linear radiometric variations or rapid evolution of geometric features, these approaches are prone to generating severe blocky artifacts or inducing spatial texture blurring, thus failing to meet the rigorous demands of high-precision monitoring [14].

In recent years, the advent of Convolutional Neural Networks (CNNs) has catalyzed significant breakthroughs in the realm of spatiotemporal fusion through data-driven approaches. In contrast to traditional paradigms, deep learning architectures possess the inherent capability to learn complex feature representations via non-linear mappings. Pioneering models, such as standard CNN-based Fusion, focused on directly learning the mapping function from low- to high-resolution domains to facilitate image fusion. Subsequently, the CTSTFM framework was introduced, employing a dual-stream architecture to independently extract temporal and spatial features. These features are then reorganized via a specialized fusion layer, effectively enhancing feature expressiveness [15]. To further exploit attention mechanisms, CAFE incorporated a feature constraint strategy, optimizing fusion weights by selectively attending to salient channels and spatial regions [16]. Moreover, Generative Adversarial Networks (GANs) have been integrated into this domain. A quintessential example is GAN-STFM, which leverages the concept of adversarial gaming. Through the adversarial training of a generator and a discriminator, it endeavors to reconstruct more realistic high-frequency texture details, thereby achieving superior perceptual quality compared to conventional models governed by Mean Squared Error (MSE) loss [13]. Furthermore, recent generative models, such as STFDiff (based on Diffusion Models) [17,18] and Mamba-STFM (based on State Space Models) [19], have demonstrated impressive capabilities in texture generation. However, they are fundamentally constrained by prohibitive computational costs and high latency. Specifically, diffusion models necessitate computationally expensive iterative denoising steps during the inference phase, rendering them impractical for the majority of remote sensing tasks that demand large-scale processing or real-time responsiveness.In addition, recent research advances have significantly expanded the scope of fusion technology, moving beyond conventional observation. Contemporary research is increasingly focusing on algorithmic perception in extreme environments, which also offers us a new perspective [20]. To provide a more intuitive comparison of these methodologies, Table 1 summarizes the core mechanisms, advantages, and primary limitations of the aforementioned representative algorithms [21,22,23,24,25].

In summary, notwithstanding the remarkable advancements achieved by deep learning-based methodologies, they remain confronted by three critical challenges:

1. Shallow Feature Interaction and the Modality Gap: The majority of existing methodologies rely primarily on rudimentary channel concatenation to integrate features from multiple sources. This shallow interaction paradigm fails to effectively bridge the inherent modality gap between heterogeneous sensors, frequently resulting in spectral reconstruction inaccuracies.

2. High-Frequency Loss Induced by Competitive Suppression: Existing spatial attention mechanisms (such as CTSTFM and CAFE) predominantly rely on simple convolution or Softmax-based normalization during the feature fusion phase. This mechanism exhibits an inherent propensity to prioritize the most salient features while suppressing secondary details. Consequently, intricate textures (such as agricultural ridges and road networks) are often “homogenized” or averaged out, resulting in a significant loss of high-frequency information.

3. Ghosting Artifacts Resulting from Insufficient Dynamic Perception: The majority of contemporary models employ static fusion rules, thereby lacking the sensitivity to perceive abrupt land surface changes (such as phenological variations). In scenarios where discrepancies exist between the reference imagery and the actual land cover at the prediction timestamp, obsolete reference information is erroneously retained, consequently introducing ghosting artifacts into the final predicted results.

## 3. Methodology

### 3.1. Problem Formulation and Overall Architecture

**Figure 1:**
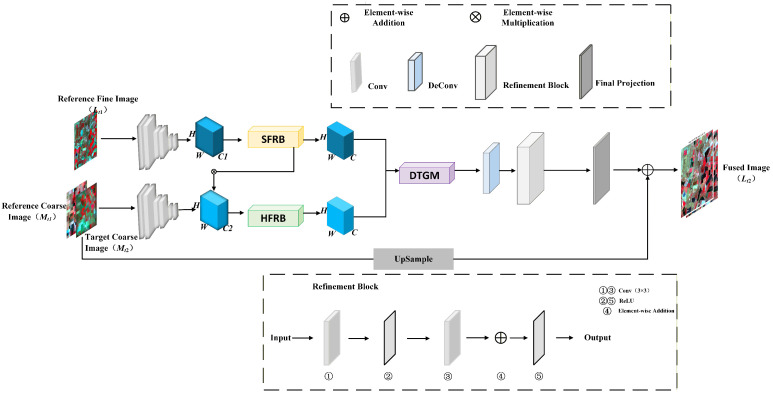
The overall architecture of the proposed XSC-Net. The network takes the reference image pair ($L_{t_{1}},M_{t_{1}}$) and the target coarse image ($M_{t_{2}}$) as inputs. It utilizes the Spectral Feature Rectification Branch (SFRB) and High-Frequency Retention Branch (HFRB) for cross-scale feature extraction, followed by the Dynamic Temporal Gating Mechanism (DTGM) to generate the final prediction $L_{t_{2}}$.

Based on the above analysis, we develop a cross-scale spectral calibration framework for spatiotemporal fusion, termed XSC-Net, which aims to mitigate spectral distortion caused by scale mismatch between coarse- and fine-resolution remote sensing images, the overall architecture of which is illustrated in Figure 1. The proposed model is primarily composed of three core components: Shallow Feature Extraction, Dual-Branch Cross-Scale Fusion, and Gated Reconstruction. Specifically, we first employ a weight-sharing convolutional encoder to extract multi-level features from both the input reference image pairs and the coarse image at the target timestamp. To simultaneously address the challenges of spectral distortion arising from heterogeneous sensors and texture over-smoothing, the extracted features are fed into two parallel processing branches: The Spectral Feature Rectification Branch (SFRB), which is designed to explicitly calibrate channel-level spectral responses by leveraging global contextual information; The High-Frequency Structure Retention Branch (HFRB), which utilizes a Sigmoid-based attention mechanism to independently preserve high-frequency spatial details. Notably, these two branches engage in deep interaction via a cross-attention strategy. Finally, the fused features are propagated into the Dynamic Temporal Gating Mechanism (DTGM). This module adaptively modulates the information flow from the reference timestamp to eliminate “ghosting” artifacts induced by land cover transitions, ultimately generating the final prediction results.

Formally, let *L* and *M* denote high-spatial-resolution (e.g., Landsat) and low-spatial-resolution (e.g., MODIS) images, respectively. The task of spatiotemporal fusion is defined as follows: Given a reference image pair at time $t_{1}$ {denoted as $\left(L_{t_{1}},M_{t_{1}}\right)$} and a coarse image at time $t_{2}$ (denoted as $M_{t_{2}}$), the objective is to predict the high-spatial-resolution image ${\hat{L}}_{t_{2}}$ at time $t_{2}$. This process can be formulated as a typical ill-posed inverse problem:

(1) $${\hat{L}}_{t_{2}}=F_{\theta }\left(L_{t_{1}},M_{t_{1}},M_{t_{2}}\right)$$

where $F_{\theta }$ denotes the proposed XSC-Net model parameterized by θ. In the subsequent subsections, we elaborate on the specific design of each core module.

### 3.2. Spectral Feature Rectification Branch

**Figure 2:**
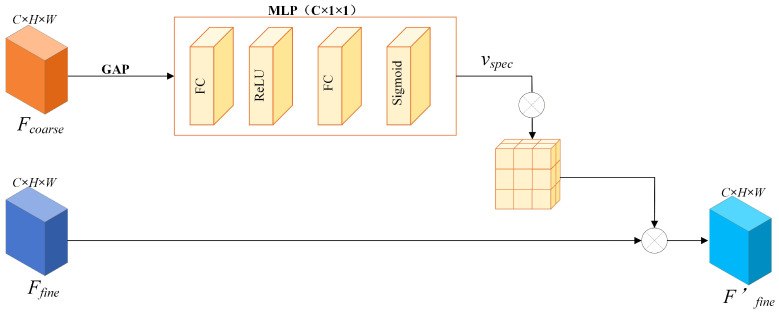
The detailed structure of the Spectral Feature Rectification Branch (SFRB). It utilizes global average pooling and an MLP to learn a calibration vector $v_{spec}$ for aligning the spectral distribution of fine features with coarse global contexts.

The SFRB is designed to leverage global contextual information from the coarse imagery to explicitly calibrate features, thereby achieving the alignment of channel-level spectral responses [26,27]. The detailed architecture of this module is illustrated in Figure 2.

Specifically, let $F_{coarse}\in R^{C\times H\times W}$ denote the input coarse feature map. Given that spectral distortion typically manifests as a global attribute, we first employ Global Average Pooling (GAP) to aggregate spatial information into a global channel descriptor $z\in R^{C\times 1\times 1}$. The *c*-th element of *z* is formulated as follows:

(2) $$z_{c}=\frac{1}{H\times W}\sum_{i=1}^{H}\sum_{j=1}^{W}F_{coarse}\left(i,j\right)$$

To explicitly model inter-channel dependencies, we employ a Multi-Layer Perceptron (MLP) mechanism comprising two Fully Connected (FC) layers (highlighted in orange in Figure 2). The spectral rectification vector $v_{spec}$ is subsequently generated as follows:

(3) $$v_{spec}=\sigma \left(W_{2}\delta \left(W_{1}z\right)\right)$$

where σ and δ denote the Sigmoid and ReLU activation functions, respectively, and $W_{1}$ and $W_{2}$ represent the weight matrices of the two fully connected layers. Through this mechanism, the network is empowered to adaptively learn the importance weights associated with distinct channels.

Upon obtaining the $v_{spec}$, as illustrated in the right panel of Figure 2, we deploy it as a “global calibration operator” applied to the fine-resolution feature map $F_{fine}\in R^{C\times H\times W}$. Specifically, we utilize a channel broadcasting mechanism to expand the vector to match the spatial dimensions of the input features, subsequently performing element-wise multiplication:

(4) $$F_{fine}^{\prime }=F_{fine}⊗v_{spec}$$

Here, ⊗ denotes element-wise multiplication, and $F_{fine}^{\prime }$ represents the fine feature map subsequent to spectral calibration. From a physical perspective, this operation is analogous to “projecting” the accurate spectral distribution inherent in the coarse imagery onto the detailed texture structure of the fine imagery.

Crucially, cross-sensor spectral consistency not only directly impacts the accuracy of the fusion results but also serves as a fundamental prerequisite for the reliable modeling of subsequent high-frequency structures. Consequently, explicitly enhancing spatial high-frequency features after completing channel-level spectral calibration holds more robust physical significance.

It is essential to clarify why the coarse-resolution image is utilized as the spectral reference to rectify the fine-resolution features. In spatiotemporal fusion, the fine-resolution image is only available at the past reference date $t_{1}$, meaning its spectral profile is outdated and cannot reflect recent temporal changes (e.g., phenological variations or floods). Conversely, the coarse-resolution image is the only reliable sensor that captures the actual spectral status at the target date $t_{2}$. Therefore, rather than calibrating the coarse image toward an outdated fine image (which would erase temporal changes), the SFRB is designed to use the coarse-resolution features as a reliable spectral anchor. This explicitly rectifies the fine-resolution features, pulling them from the $t_{1}$ spectral domain into the correct $t_{2}$ spectral domain while preserving their spatial structural advantages.

### 3.3. High-Frequency Retention Branch

**Figure 3:**
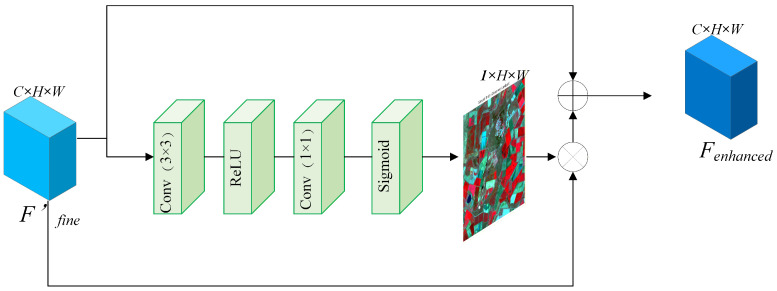
Thedetailed structure of the High-Frequency Retention Branch (HFRB). By employing a non-competitive Sigmoid activation, it generates a spatial attention mask $M_{spatial}$ to independently activate and preserve high-frequency details such as edges and boundaries.

To circumvent the “over-smoothing” problem induced by traditional Softmax attention mechanisms, we devise the High-Frequency Structure Retention Block (HFRB). This module employs a Sigmoid-based non-competitive activation strategy to independently preserve high-frequency spatial details. The specific architecture of this block is depicted in Figure 3.

Specifically, the HFRB receives the features $F_{fine}^{\prime }$ calibrated by the SFRB as input. To generate a spatial attention mask capable of capturing high-frequency details [28], we first extract spatial features via a convolutional layer. Subsequently, eschewing the conventional Softmax activation, we utilize the Sigmoid function to generate a non-competitive probability map $M_{spatial}\in R^{1\times H\times W}$ (refer to the green slice in Figure 3):

(5) $$M_{spatial}=\sigma \left({\mathrm{Conv}}_{1\times 1}\left(\delta \left({\mathrm{Conv}}_{3\times 3}\left(F_{fine}^{\prime }\right)\right)\right)\right)$$

Here, the Sigmoid function permits each pixel within the attention mask to independently output a response value in the range of [0, 1]. This implies that within complex heterogeneous regions, multiple adjacent pixels (e.g., dense building edges) can simultaneously achieve high response values. This phenomenon, which we characterize as a “multi-hot” property, effectively circumvents the feature suppression caused by the sum-to-one constraint inherent in Softmax.

Finally, we modulate the input features using the generated spatial attention mask and integrate them via a residual connection [29,30,31] to yield the enhanced features $F_{enhanced}$:

(6) $$F_{enhanced}=F_{fine}^{\prime }+\left(F_{fine}^{\prime }⊗M_{spatial}\right)$$

By virtue of this design, the HFRB effectively enhances high-frequency texture information, thereby endowing the final fused imagery with sharp geometric details.

It is worth noting that while our SFRB and HFRB may structurally resemble generic attention mechanisms like SE-Net and CBAM, their fundamental design motivations are strictly task-specific for spatiotemporal fusion. Generic attention mechanisms primarily perform internal feature reweighting blindly to locate salient objects. In contrast, our SFRB acts as a physical cross-scale calibrator, explicitly leveraging the spectral fidelity of the coarse image to guide the rectification of the fine image. Furthermore, traditional spatial attention heavily relies on pooling operations that inevitably smoothting structural details. To overcome this, our HFRB functions as a high-frequency detail injector, specifically tailored to preserve and transfer sub-pixel boundaries and textures from the high-resolution reference, effectively resolving the cross-scale spectral inconsistency and detail degradation problems.

### 3.4. Dynamic Temporal Gating Mechanism

**Figure 4:**
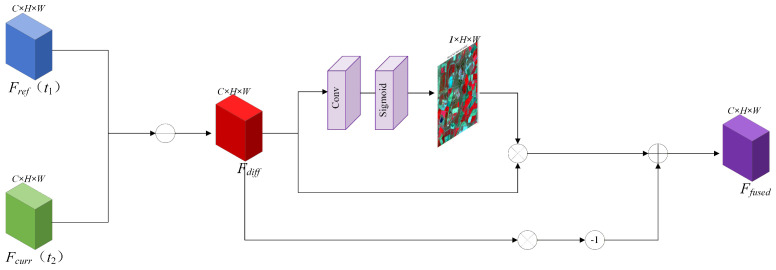
The detailed structure of the Dynamic Temporal Gating Mechanism (DTGM). It computes the difference feature $F_{diff}$ to generate a dynamic gate map *G*, adaptively fusing the reference and current features to mitigate ghosting artifacts caused by land cover changes.

In spatiotemporal fusion tasks, abrupt temporal land cover changes (such as crop harvesting or flood inundation) constitute the primary cause of “ghosting” artifacts. To effectively address this challenge, we propose the Dynamic Temporal Gating Mechanism (DTGM), the structure of which is illustrated in Figure 4.

Positioned at the final stage of the feature fusion process, this module is responsible for dynamically modulating the fusion ratio between the information from the reference timestamp and that of the prediction timestamp. Let $F_{ref}$ denote the reference features at time $t_{1}$, and $F_{curr}$ represent the features at time $t_{2}$ predicted by the current branch. The DTGM first computes the absolute difference map between these two components (refer to the left panel of Figure 4) to detect potential regions of change:

(7) $$F_{diff}=\left|F_{curr}-F_{ref}\right|$$

Subsequently, these difference features $F_{diff}$ are fed into a convolution-based gating network to generate a dynamic gating map $G\in R^{1\times H\times W}$. The values of this map are constrained to the range [0,1], serving to quantify the probability of change occurrence at each individual pixel:

(8) $$G=\sigma (\mathrm{Conv}\left(F_{diff}\right))$$

The final fused features $F_{fused}$ are derived via the following weighting formulation:

(9) $$F_{fused}=G⊗F_{curr}+\left(1-G\right)⊗F_{ref}$$

The physical interpretation of this formula is highly intuitive: when the gating value *G* approaches 1, it indicates that significant changes have occurred within the region, prompting the model to rely predominantly on the currently predicted features $F_{curr}$. Conversely, when the value *G* approaches 0, it suggests the temporal stability of the land cover, leading the model to maximize the utilization of high-frequency details $F_{ref}$ from the reference timestamp.

A common concern in spatiotemporal fusion is whether temporal changes derived from coarse-resolution images can adequately reflect small-scale (sub-pixel) land-cover variations. It is important to note that while coarse pixels lack the spatial resolution to explicitly delineate the structural boundaries of small changes, they possess high radiometric sensitivity to capture the overall spectral energy shifts caused by these variations. In our architecture, the temporal difference extracted by DTGM serves strictly as an adaptive modulator (gating weight). By synergizing the temporally sensitive weights from DTGM with the high-frequency spatial priors preserved by the HFRB module, the deep network effectively maps the subtle sub-pixel spectral variations in the coarse images to fine-grained structural changes in the final high-resolution output.

### 3.5. Reconstruction and Loss Function

The feature maps fused via the DTGM are subsequently fed into the decoder module (refer to the decoding section on the right side of Figure 1). This decoder comprises a cascade of deconvolutional layers and convolutional refinement blocks, designed to progressively upsample the deep features and map them back to the original image space, ultimately generating the high-resolution predicted image ${\hat{L}}_{t_{2}}$ at time $t_{2}$.

To supervise the parameter optimization of the entire XSC-Net framework, we define an objective function to quantify the discrepancy between the predicted results and the ground truth. In this study, we employ the $L_{1}$ norm (Mean Absolute Error, MAE) as the loss function, aimed at minimizing the pixel-wise errors between the predicted image ${\hat{L}}_{t_{2}}$ and the actual ground truth image $L_{t_{2}}$.

The preference for the $L_{1}$ loss over the prevalent $L_{2}$ loss (Mean Squared Error) is grounded in two primary rationales: (1) The $L_{2}$ loss tends to disproportionately penalize larger errors, a characteristic that often induces an “over-smoothing” or blurring effect on textural details [13]. In contrast, the $L_{1}$ loss exhibits superior convergence properties regarding edges and high-frequency details, thereby facilitating the generation of sharper structural boundaries. (2) The $L_{1}$ loss demonstrates inherent robustness against outliers, a critical attribute for mitigating the impact of transient noise frequently encountered in remote sensing imagery.

The loss function is mathematically formulated as follows:

(10) $$L=\frac{1}{N}\sum_{i=1}^{N}{∥{\hat{L}}_{t_{2}}^{i}-L_{t_{2}}^{i}∥}_{1}$$

where *N* denotes the batch size, and ${∥\cdot ∥}_{1}$ represents the $L_{1}$ norm. During the training phase, this loss is minimized via the backpropagation algorithm to optimize the network parameters in an end-to-end manner.

It is worth noting that our training objective relies solely on the $L_{1}$ reconstruction loss. While recent vision tasks often employ complex hybrid losses (e.g., perceptual or adversarial losses), they are typically formulated for standard RGB images and may introduce unpredictable spectral artifacts when applied to multispectral remote sensing data. Furthermore, unlike models that rely on complex loss functions to force detail recovery, our proposed architecture explicitly addresses detail preservation at the feature level through the HFRB module. Consequently, the simple $L_{1}$ loss is mathematically sufficient to drive the network towards stable convergence while effectively preserving spatial boundaries and preventing the over-smoothing effect often caused by $L_{2}$ loss.

## 4. Results

### 4.1. Datasets

To comprehensively evaluate the performance of XSC-Net, we employed two public benchmark datasets exhibiting distinct land surface characteristics: the Coleambally Irrigation Area (CIA) and the Lower Gwydir Catchment (LGC) [32]. Situated in New South Wales, Australia, both datasets encompass rich phenological variations and abrupt land cover transitions.

**(1) Coleambally Irrigation Area (CIA):** The CIA dataset is characterized predominantly by highly heterogeneous agricultural landscapes, featuring a multitude of small, irregularly shaped crop patches. The dataset spans from October 2001 to May 2002, encompassing the complete phenological cycle of crops from the growing season to harvest. For this study, we utilized 17 pairs of cloud-free Landsat-MODIS imagery. Due to the high degree of landscape fragmentation in this region, it imposes stringent demands on the model’s capacity to capture high-frequency spatial details (agricultural ridges and crop boundaries). Consequently, this dataset serves as an ideal benchmark for validating the efficacy of our proposed HFRB.
**(2) Lower Gwydir Catchment (LGC):** The LGC dataset primarily covers extensive areas of homogeneous agricultural land and natural vegetation. Its temporal span ranges April 2004 to early 2005, comprising 14 image pairs. In contrast to the CIA, the LGC dataset captures a large-scale flood event that occurred in December 2004, which induced significant abrupt transitions in land cover. Such drastic non-linear variations present an ideal scenario for benchmarking the model’s capability to dynamically capture temporal changes.

### 4.2. Evaluation Metrics

To conduct a multidimensional assessment of the XSC-Net performance—specifically encompassing spectral fidelity, structural preservation, and global reconstruction error—we employed four quantitative metrics widely accepted in the field of spatiotemporal fusion: Root Mean Square Error (RMSE), Peak Signal-to-Noise Ratio (PSNR), Structural Similarity Index (SSIM), and Spectral Angle Mapper (SAM).

**(1) Root Mean Square Error (RMSE):** The RMSE serves as the most intuitive metric for quantifying the disparity between the fused imagery and the ground truth [33]. It computes the square root of the mean squared pixel-wise differences between the predicted and actual values, thereby reflecting the global radiometric deviation of the imagery. A lower RMSE value indicates a closer approximation to the ground truth, signifying higher prediction accuracy. The mathematical formulation is defined as:
  (11) $$\mathrm{RMSE}=\sqrt{\frac{1}{H\times W\times C}\sum_{k=1}^{C}\sum_{i=1}^{H}\sum_{j=1}^{W}{\left({\hat{L}}_{t_{2}}\left(i,j,k\right)-L_{t_{2}}\left(i,j,k\right)\right)}^{2}}$$
  where *H*, *W*, and *C* denote the height, width, and the number of spectral bands of the imagery, respectively; while k,i,j serve as the indices corresponding to the spectral band, row (height), and column (width). Furthermore, ${\hat{L}}_{t_{2}}$ and $L_{t_{2}}$ represent the pixel values of the predicted image and the ground truth reference image.
**(2) Peak Signal-to-Noise Ratio (PSNR):** PSNR is an image quality evaluation metric based on error sensitivity, employed to quantify the extent of signal distortion during the image reconstruction process [33]. It assesses image quality by computing the ratio between the maximum possible power of the signal and the power of corrupting noise. Measured in decibels (dB), a higher PSNR value indicates reduced image distortion and superior recovery of spatial details. Generally, values exceeding 30 dB are indicative of high-fidelity image quality. The calculation formula is defined as follows:
  (12) $$\mathrm{PSNR}=10\cdot \log_{10}\left(\frac{{\mathrm{MAX}}_{I}^{2}}{\mathrm{MSE}}\right)$$
  where MSE denotes the Mean Squared Error, and ${\mathrm{MAX}}_{I}$ represents the maximum attainable pixel intensity of the image (given that the experimental data has been normalized to the [0, 1] interval, ${\mathrm{MAX}}_{I}=1$ in this context).
**(3) Structural Similarity Index (SSIM):** Distinct from pixel-error-based metrics such as RMSE and PSNR, the SSIM is a perception-based metric designed to simulate the sensitivity of the Human Visual System (HVS) to structural information [34]. It assesses the similarity between two images across three independent dimensions: luminance, contrast, and structure. The index ranges from [−1, 1]; a value approaching 1 indicates a higher consistency between the fused imagery and the ground truth in terms of geometric structure and textural edges. Consequently, this metric serves as an effective indicator of the model’s capacity for preserving geometric structures and local textures. The formula is defined as:
  (13) $$\mathrm{SSIM}\left(x,y\right)=\frac{\left(2\mu_{x}\mu_{y}+C_{1}\right)\left(2\sigma_{xy}+C_{2}\right)}{\left(\mu_{x}^{2}+\mu_{y}^{2}+C_{1}\right)\left(\sigma_{x}^{2}+\sigma_{y}^{2}+C_{2}\right)}$$
  where $\mu_{x}$ and $\mu_{y}$ denote the mean intensities of images *x* and *y*, $\sigma_{x}^{2}$ and $\sigma_{y}^{2}$ represent the variances, and $\sigma_{xy}$ denotes the covariance. To mitigate numerical instability arising from a zero denominator, constants $C_{1}={\left(k_{1}L\right)}^{2}$ and $C_{2}={\left(k_{2}L\right)}^{2}$ are introduced. Here, *L* signifies the dynamic range of the pixel values (since the imagery has been previously normalized, L=1), while $k_{1}=0.01$ and $k_{2}=0.03$ serve as default constants.
**(4) Spectral Angle Mapper (SAM):** SAM is primarily employed to assess the spectral fidelity of multispectral remote sensing imagery. It conceptually models the spectrum of each pixel as an *C*-dimensional vector, quantifying spectral distortion by computing the angle between the predicted spectral vector and the ground truth spectral vector [35,36]. SAM is typically expressed in degrees or radians. A smaller SAM value (approaching 0) denotes a higher directional consistency between the two vectors, signifying that the spectral characteristics of the predicted image are free from significant spectral shifts. Consequently, this metric serves as a direct indicator of the superiority of our proposed SFRB module in the domain of spectral calibration. The formula is presented as follows:
  (14) $$\mathrm{SAM}=\frac{1}{H\times W}\sum_{i=1}^{H}\sum_{j=1}^{W}\arccos\left(\frac{\langle v_{i,j},{\hat{v}}_{i,j}\rangle }{{∥v_{i,j}∥}_{2}\cdot {∥{\hat{v}}_{i,j}∥}_{2}}\right)$$
  where i,j serves as the spatial coordinate index of the pixel; $v_{i,j}$ and ${\hat{v}}_{i,j}$ denote the predicted spectral vector and the ground truth spectral vector located at (i,j). Furthermore, 〈·,·〉 represents the vector dot product, and ${∥\cdot ∥}_{2}$ signifies the $L_{2}$ norm.

### 4.3. Implementation Details

The proposed XSC-Net is implemented using the PyTorch 1.11.0 deep learning framework, operating within a Python 3.8 and CUDA 11.3 environment. All experiments were conducted on a high-performance computing platform equipped with an Intel(R) Xeon(R) Platinum 8470Q processor (20 vCPUs) and a single NVIDIA GPU (vGPU) with 48 GB of video memory. This hardware configuration provides robust computational support for the efficient processing of large-scale remote sensing datasets.

To train the proposed deep learning models, the original large-scale Landsat and MODIS remote sensing images from both the CIA and LGC datasets were spatially cropped into small overlapping patches of size 40 × 40. The generated datasets were strictly divided into a training set and a testing set with a ratio of 8:2. Specifically, for the CIA dataset, the total 35,088 generated patches were divided into 28,070 patches for training and 7018 patches for testing. Similarly, for the LGC dataset, the total 70,720 patches were divided into 56,576 training patches and 14,144 testing patches. To comprehensively evaluate the efficiency of the proposed method, we calculated the computational complexity of the models. Our model requires only 2.75 M parameters, requires only 11.73 G FLOPs. Furthermore, the average inference time during the testing phase is approximately 0.07 s/patch.

During the data preprocessing stage, all spectral band values were normalized to stabilize the network convergence. In the training phase, the network was optimized using the Adam optimizer with a batch size of 64 for 300 epochs. As shown in the training configuration, learning rate decay was strategically applied to ensure optimal convergence. Furthermore, to strictly preserve the geographical orientation and spatial structures of the remote sensing data, no dynamic data augmentation techniques were applied during training.

### 4.4. Comparison with State-of-the-Arts Methods

To conduct a comprehensive and fair evaluation of XSC-Net, we benchmarked it against seven algorithms that represent either significant milestones or state-of-the-art (SOTA) techniques within the domain of spatiotemporal fusion. These selected baselines encompass a broad spectrum of methodologies, ranging from traditional weight-based and hybrid models to advanced deep learning approaches incorporating Convolutional Neural Network (CNN), Generative Adversarial Network (GAN), and attention mechanisms, thereby ensuring wide representativeness. The specific comparative methods are detailed as follows:

- **STARFM [8]:** Recognized as a pioneering algorithm in this domain, STARFM is grounded in spectral mixing theory. It operates on the premise that the spectral variations of coarse and fine-resolution imagery are consistent for identical land cover types over time. By leveraging spectrally similar pixels within a sliding window to compute the weighted prediction of the central pixel, STARFM serves as a classic benchmark for assessing fusion performance.
- **ESTARFM [9]:** Designed to address the deficiencies of STARFM in heterogeneous landscapes, ESTARFM incorporates conversion coefficients to effectively manage the mixed pixel problem. This method necessitates two pairs of reference images and is specifically aimed at enhancing prediction accuracy in scenarios characterized by complex land cover changes.
- **FSDAF [10]:** As a quintessential hybrid approach, FSDAF ingeniously integrates spectral unmixing with Thin Plate Spline (TPS) interpolation. Capable of capturing both gradual variations and abrupt events using a single pair of reference images, it stands as one of the most robust representatives among traditional methodologies.
- **GAN-STFM [13]:** This method pioneers the integration of Generative Adversarial Network (GAN) into the domain of spatiotemporal fusion. By leveraging the adversarial interplay between a generator and a discriminator, GAN-STFM endeavors to reconstruct realistic high-frequency textural details. Consequently, it typically exhibits superior visual perceptual quality compared to models optimized solely on pixel-wise error metrics.
- **CTSTFM [15]:** Representing an early iteration of a two-stream CNN architecture, this model employs two distinct branches to independently extract temporal and spatial features, which are subsequently recombined via a fusion layer. It signifies a preliminary exploration of deep learning capabilities regarding automatic feature extraction.
- **CAFE [16]:** This method incorporates an attention mechanism to augment feature representation. By prioritizing salient channel and spatial regions, CAFE aims to mitigate the issue of inefficient feature utilization inherent in traditional CNNs, serving as a prominent exemplar of attention-based methodologies in this field.
- **SDCS [37]:** Furthermore, to comprehensively evaluate the superiority of our proposed model against the most recent advancements, we incorporated SDCS, a highly representative deep learning-based spatiotemporal fusion method published in 2024. By establishing a quantitatively descriptive framework, this method demonstrates strong feature recovery capabilities and serves as the latest state-of-the-art baseline in our comparative experiments.

### 4.5. Quantitative Analysis and Visual Results

**Figure 5:**
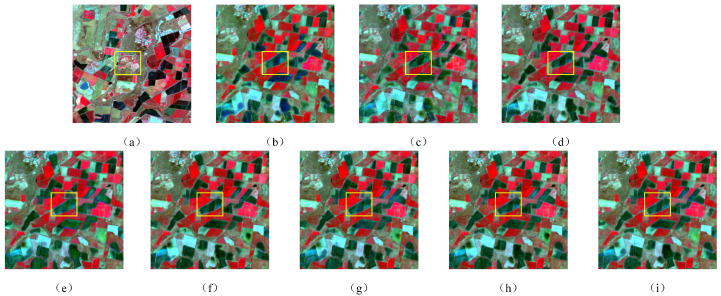
Visual comparison of fusion results on a heterogeneous sub-region of the CIA dataset. (**a**) Ground Truth ($L_{t_{2}}$). (**b**) STARFM. (**c**) ESTARFM. (**d**) FSDAF. (**e**) GAN-STFM. (**f**) CAFE. (**g**) CTSTFM. (**h**) SDCS. (**i**) XSC-Net.

To comprehensively validate the efficacy of XSC-Net, we conducted a comprehensive comparative analysis against three representative traditional algorithms (STARFM, ESTARFM, and FSDAF) and three advanced deep learning approaches (GAN-STFM, CAFE, and CTSTFM). Table 2 presents the quantitative performance metrics for all competing methods on the CIA dataset, while Figure 5 illustrates the local visual reconstruction results over representative heterogeneous regions.

As evidenced by the quantitative metrics presented in Table 2, XSC-Net achieved either the best or second-best performance across all four indices—RMSE, SAM, PSNR, and SSIM—thereby demonstrating substantial superiority. To facilitate the interpretation of the experimental results, the best values within the table are highlighted in bold, while the second-best values are indicated by underlining.

Traditional methods, such as STARFM and FSDAF, often struggle to capture the non-linear variations of complex land covers due to their reliance on linear spectral mixing assumptions. Although FSDAF is widely regarded as the most robust among traditional approaches, XSC-Net significantly outperforms it, achieving a PSNR improvement of 5.43 dB and a 30% reduction in RMSE (decreasing from 0.023 to 0.016). This substantial margin provides compelling evidence of the superior non-linear mapping capability driven by deep learning. When benchmarked against state-of-the-art deep learning counterparts, XSC-Net exhibits similarly outstanding performance. Attributable to the precise spectral channel calibration facilitated by the SFRB module, XSC-Net achieves a PSNR of 36.63 dB, surpassing the runner-up method, CTSTFM (35.03 dB), by 1.60 dB. This indicates a highly effective mitigation of spectral distortion arising from heterogeneous sensors. Furthermore, the recorded SSIM score of 0.931 highlights the model’s excellence in maintaining structural integrity and luminance contrast. This result serves as empirical evidence of the HFRB efficacy in preserving high-frequency spatial information. It is worth noting that SDCS, as the most recently proposed baseline (2024), exhibits highly competitive overall performance, generally outperforming earlier methods. However, as illustrated in Table 2, our proposed XSC-Net consistently surpasses SDCS across both the CIA and LGC datasets. Specifically, XSC-Net maintains a significant lead in the SSIM metric, which intrinsically measures structural similarity and high-frequency edge preservation capabilities. This result compellingly demonstrates that XSC-Net achieves the greatest performance, even when compared with the most advanced spatiotemporal fusion algorithms from the past two years.

This conclusion is further corroborated by the visual qualitative comparison presented in Figure 5. To clearly delineate vegetation growth status and land cover details, all experimental results are visualized using a Standard False-Color Composition (NIR-Red-Green). Observations from Figure 5 reveal that traditional methods—particularly STARFM—are constrained by their sliding window mechanisms, exhibiting distinct blocky artifacts and textural blurring during the reconstruction process. While FSDAF and ESTARFM demonstrate improvements, significant spectral mixing persists within heterogeneous regions situated at the boundaries of different crops. In contrast, while existing deep learning methods enhance overall clarity, they struggle to simultaneously balance detail preservation and artifact suppression: GAN-STFM introduces unnatural noise in certain areas due to the instability inherent in adversarial training, whereas CAFE tends towards over-smoothing, resulting in the loss of fine textures such as field ridges.

Conversely, the reconstruction results yielded by our XSC-Net exhibit a high degree of visual consistency with the ground truth. Attributable to the non-competitive activation strategy of the HFRB, XSC-Net not only generates sharp field boundaries and distinct ridge lines but also effectively eliminates ghosting artifacts and chromatic deviations. It maintains superior spectral fidelity while simultaneously preserving high-frequency geometric details. In summary, in terms of both objective quantitative metrics and subjective visual assessments, XSC-Net consistently outperforms existing state-of-the-art methods, thereby validating the effectiveness of our proposed cross-scale spectral calibration strategy in spatiotemporal fusion tasks.

**Figure 6:**
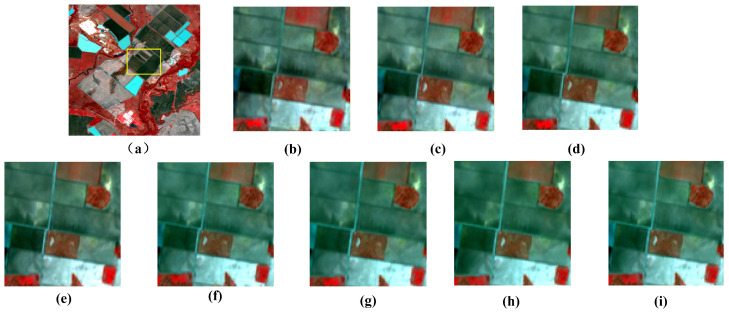
Visual comparison of fusion results on a heterogeneous sub-region of the LGC dataset. (**a**) Ground Truth ($L_{t_{2}}$). (**b**) STARFM. (**c**) ESTARFM. (**d**) FSDAF. (**e**) GAN-STFM. (**f**) CAFE. (**g**) CTSTFM. (**h**) SDCS. (**i**) XSC-Net.

To further validate the generalization capability of XSC-Net across diverse geographical environments and land cover types, we conducted supplementary evaluation experiments on the Lower Gwydir Catchment (LGC) dataset. In contrast to the CIA dataset, which is characterized primarily by regularly distributed farmland changes, the LGC dataset encompasses substantial natural vegetation and water body variations induced by flooding, thereby exhibiting a higher degree of spatiotemporal heterogeneity. Table 3 presents the quantitative evaluation metrics for all methods on the LGC dataset. Given space constraints and the high consistency of the visual improvements with those observed in the CIA dataset, we report solely the quantitative results for the LGC dataset in this section. Figure 6 shows the local reconstruction results of each method on the LGC dataset.

To further evaluate the model’s performance in handling abrupt land-cover changes, we present visual comparison results on the LGC dataset in Figure 6. This dataset captures a large-scale flood event, where complex flood boundaries pose significant challenges for spatiotemporal fusion. As illustrated in the magnified regions of Figure 6, traditional methods such as STARFM and ESTARFM suffer from severe blurring and fail to recover clear flood boundaries. Although deep learning-based approaches, including CTSTFM and CAFE, show some improvement in visual clarity, they still exhibit varying degrees of oversmoothing. In contrast, the proposed XSC-Net produces fusion results that most closely approximate the ground truth, effectively preserving complex boundaries and fine-grained spatial details within the flooded regions. These visual results provide strong evidence of XSC-Net’s robustness in handling abrupt temporal changes.

In comparison to the traditional FSDAF method, XSC-Net elevates the PSNR from 31.55 dB to 35.82 dB, marking an improvement exceeding 4 dB. This demonstrates that even when confronting scenarios characterized by irregular land cover geometries and drastic spectral variations—such as those in the LGC dataset—the proposed non-linear mapping network maintains robust stability. Furthermore, on the LGC dataset, XSC-Net achieved a SAM value of 3.015, indicating that the imagery generated by our model most closely approximates the ground truth in the spectral dimension. It accurately captures the spectral characteristic transitions of vegetation and water bodies pre- and post-flood, without suffering from spectral distortions induced by complex background interference. In conclusion, the experimental results on the LGC dataset align closely with those from the CIA dataset, compellingly demonstrating that XSC-Net not only performs exceptionally in regular farmland scenarios but also possesses superior generalization capability and practical utility in complex natural landscapes.

### 4.6. Ablation Study

To rigorously validate the efficacy of the core modules embedded within XSC-Net, we established a network comprising solely a basic convolutional encoder–decoder as the Baseline model. Subsequently, we conducted an ablation study on the CIA dataset, characterized by the incremental integration of specific modules. Table 4 presents a detailed quantitative evaluation of the various model variants.

As illustrated in Table 4, the incorporation of the Spectral Feature Refinement Block (SFRB) in Model-A yielded significant improvements across all metrics compared to the Baseline. Notably, the SAM metric experienced a substantial reduction from 3.520 to 3.153, while the PSNR increased by 1.27 dB. This provides direct evidence that the SFRB, by calibrating channel responses via global context, effectively mitigates the spectral distortion issues inherent in heterogeneous sensors, thereby significantly enhancing the spectral fidelity of the fused imagery.

The subsequent integration of the High-Frequency Retention Block (HFRB) into Model-A (forming Model-B) primarily yielded improvements in structural similarity metrics. Specifically, the SSIM rose from 0.902 to 0.925, accompanied by a further reduction in RMSE. These results demonstrate that the Sigmoid-based non-competitive activation mechanism embedded within the HFRB successfully captured enriched edge and texture details—such as field ridges and roads—thereby effectively mitigating the texture over-smoothing phenomenon commonly observed in traditional convolutional networks.

Finally, with the integration of the Dynamic Temporal Gating Mechanism (constituting the complete XSC-Net), the model achieved optimal performance. Although the numerical improvements over Model-B were relatively marginal, this step is critical for eliminating “ghosting” artifacts induced by moving objects or land cover changes, thereby ensuring the temporal logical accuracy of the generated imagery. In summary, the ablation study provides compelling evidence that the three core components of XSC-Net play indispensable roles in enhancing spectral fidelity, preserving spatial details, and suppressing temporal artifacts, respectively.

**Figure 7:**
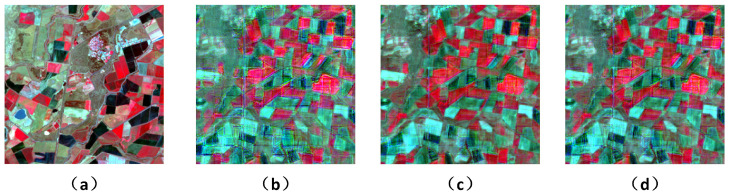
Visual comparison of the ablation study. (**a**) Ground Truth ($L_{t_{2}}$). (**b**) Baseline. (**c**) Model with SFRB. (**d**) Model with SFRB and HFRB.

To intuitively verify the validity of our proposed modules and corroborate the quantitative results, we further provide a visual comparison of different network variants in Figure 7. As shown in the figure, emoving the SFRB leads to distinct spectral inconsistency, failing to reliably match the color distribution of the ground truth. Furthermore, discarding the HFRB results in overly smoothed spatial boundaries and the severe degradation of fine-grained structures. In contrast, the full XSC-Net effectively overcomes these limitations, achieving the highest visual fidelity in preserving both spectral properties and high-frequency spatial details. This visual evidence strictly aligns with the quantitative metrics presented in Table 4, thoroughly confirming the indispensability of the proposed modules.

To explicitly validate the superiority of our tailored modules over generic attention mechanisms, we conducted a comprehensive ablation study by replacing the proposed SFRB and HFRB with the widely used SE-Net and CBAM, respectively. The quantitative results of different module combinations on the CIA dataset are presented in Table 5.

As observed, the baseline configuration utilizing both generic SE-Net and CBAM yields sub-optimal performance. When substituting SE-Net with our proposed SFRB, the SAM metric improves significantly. This confirms that SFRB effectively mitigates the severe spectral distortion caused by the self-attention in SE-Net. Conversely, substituting CBAM with our HFRB leads to a substantial gain in the SSIM metric. This clearly demonstrates the HFRB’s superior capability in preserving high-frequency spatial structures, successfully avoiding the over-smoothing penalty inherently caused by the global pooling operations in CBAM. Finally, the complete XSC-Net, which integrates both SFRB and HFRB, achieves the best overall performance across all evaluation metrics. This explicitly proves the indispensability and complementarity of the proposed modules for capturing both accurate spectral fidelity and sharp spatial details.

To explicitly validate our claim regarding the activation function, we compared the network performance using Softmax versus Sigmoid. As demonstrated in Table 6, the Sigmoid function significantly outperforms Softmax.

As shown in Table 6, replacing Softmax with Sigmoid yields significant improvements, particularly in SSIM. As a metric for measuring the similarity of image structure and edge texture, SSIM improved from 0.903 to 0.931 after using Sigmoid.

## 5. Discussion

In this paper, we propose XSC-Net, a novel Cross-scale Spectral Calibration Network designed for the spatiotemporal fusion of remote sensing imagery. To address the challenges of spectral distortion and texture over-smoothing inherent in existing deep learning methodologies, we introduce a unified framework that synergizes spectral calibration with high-frequency retention. Specifically, through the synergistic design of cross-scale spectral calibration and non-competitive high-frequency enhancement, XSC-Net effectively mitigates the persistent problems of spectral distortion and structural degradation in the field of deep learning-based spatiotemporal fusion.

Extensive experiments conducted on both the CIA and LGC datasets demonstrate XSC-Net’s superiority over numerous current state-of-the-art methods. Quantitatively, our model achieved the lowest prediction error (RMSE) and the highest structural similarity (SSIM), attaining a PSNR of 36.63 dB on the CIA dataset. Qualitatively, the visual results corroborate that the fused images generated by XSC-Net possess sharper textures and more accurate spectral attributes.

## 6. Conclusions

Despite the superior spatiotemporal fusion performance achieved by the proposed XSC-Net on the widely adopted CIA and LGC benchmarks, there are still some limitations to be addressed. For instance, further extensive validation across more diverse global ecological regions—such as tropical rainforests or dense urban areas—is needed to comprehensively evaluate its generalization capabilities. Second, similar to most existing fusion frameworks, our model heavily relies on the availability of high-quality, cloud-free reference image pairs, whereas acquiring completely clear optical images in practical scenarios is often hindered by frequent cloud cover. In future work, we plan to construct more diverse and large-scale spatiotemporal fusion datasets to further validate and optimize our framework. 

## Figures and Tables

**Table 1:** Summary of representative spatiotemporal fusion algorithms, their key mechanisms, pros, and cons.

Algorithm	Category	Key Mechanism	Pros	Cons
STARFM	Weight-based	Spectral similarity weighting	First of its kind	Blocky artifacts; Linear assumption
ESTARFM	Weight-based	Conversion coefficients	Handles heterogeneity	Computationally costly; Needs two pairs
FSDAF	Hybrid	Unmixing + TPS interpolation	Needs only one pair; Robust	Blurring in transition zones
CTSTFM	Deep Learning	Two-stream CNN	Non-linear mapping	Spectral distortion; Shallow interaction
CAFE	Deep Learning	Attention mechanism	Feature constraints	Over-smoothed details; Competitive suppression
GAN-STFM	GAN-based	Adversarial training	Sharp textures	Training instability; Hallucinations

**Table 2:** Quantitative comparison of different fusion methods on the CIA dataset. The best results are highlighted in **bold**, and the second-best results are underlined.

	STARFM	FSDAF	ESTARFM	GAN-STFM	CAFE	CTSTFM	SDCS	XSC-Net
RMSE (↓)	0.027	0.023	0.025	0.018	0.019	0.017	0.018	**0.016**
SAM (↓)	5.775	5.051	3.950	3.101	3.575	3.233	3.022	**2.948**
PSNR (↑)	29.85	31.20	30.50	34.52	34.35	35.03	34.69	**36.63**
SSIM (↑)	0.868	0.851	0.835	0.905	0.911	0.901	0.909	**0.931**

**Table 3:** Quantitative comparison of different fusion methods on the LGC dataset for generalization analysis. The best results are highlighted in **bold**, and the second-best results are underlined.

	STARFM	FSDAF	ESTARFM	GAN-STFM	CAFE	CTSTFM	SDCS	XSC-Net
RMSE (↓)	0.028	0.024	0.026	0.021	0.020	0.017	0.019	**0.014**
SAM (↓)	3.842	3.415	3.650	3.280	3.284	3.122	3.233	**3.015**
PSNR (↑)	29.15	31.55	30.02	33.20	33.68	34.85	35.32	**35.82**
SSIM (↑)	0.865	0.892	0.878	0.904	0.901	0.912	0.914	**0.928**

**Table 4:** Ablation study on the effectiveness of different modules in XSC-NET. “√” indicates the inclusion of the corresponding component.

Model	SFRB	HFRB	DTGM	RMSE	PSNR	SSIM	SAM
Baseline	×	×	×	0.021	33.85	0.884	3.520
Model-A	√	×	×	0.019	35.12	0.902	3.151
Model-B	√	√	×	0.017	36.15	0.925	3.013
XSC-Net	√	√	√	0.016	36.63	0.931	2.948

**Table 5:** Quantitative comparison of different spectral and spatial module combinations on the CIA dataset.

Model			RMSE	PSNR	SSIM	SAM
Model-A	SE-Net	CBAM	0.025	33.85	0.884	3.623
Model-B	SFRB	CBAM	0.023	34.12	0.897	3.322
Model-C	SE-Net	HFRB	0.019	35.86	0.925	3.226
XSC-Net	SFRB	HFRB	0.016	36.63	0.931	2.948

**Table 6:** Quantitative Comparison of Different Activation Functions on the CIA Dataset.

	RMSE	PSNR	SSIM	SAM
Softmax	0.021	35.96	0.903	3.122
Sigmoid	0.016	36.63	0.931	2.948

## Data Availability

The CIA and LGC datasets used in this study are publicly available at references. The code presented in this study is available on request from the corresponding author.

## References

[1] Wulder M.A., Roy D.P., Radeloff V.C., Loveland T.R., Anderson M.C., Johnson D.M., Healey S., Zhu Z., Scambos T.A., Pahlevan N. (2022). Fifty years of Landsat science and impacts. Remote Sens. Environ..

[2] Irons J., Dwyer J.L., Barsi J.A. (2012). The next Landsat satellite: The Landsat data continuity mission. Remote Sens. Environ..

[3] Small C. (2004). The Landsat ETM+ spectral mixing space. Remote Sens. Environ..

[4] Zhu Z., Woodcock C.E. (2014). Automated cloud, cloud shadow, and snow detection in multitemporal Landsat data: An algorithm designed specifically for monitoring land cover change. Remote Sens. Environ..

[5] Shen H., Li X., Cheng Q., Zeng C., Yang G., Li H., Zhang L. (2015). Missing Information Reconstruction of Remote Sensing Data: A Technical Review. IEEE Geosci. Remote Sens..

[6] Lian Z., Zhan Y., Zhang W., Wang Z., Liu W., Huang X. (2025). Recent advances in deep learning-based spatiotemporal fusion methods for remote sensing images. Sensors.

[7] Sun E., Cui Y., Liu P., Yan J. (2025). A decade of deep learning for remote sensing spatiotemporal fusion: Advances, challenges, and opportunities. IEEE Geosci. Remote Sens. Mag..

[8] Gao F., Masek J., Schwaller M., Hall F. (2006). On the blending of the Landsat and MODIS surface reflectance: Predicting daily Landsat surface reflectance. IEEE Trans. Geosci. Remote Sens..

[9] Zhu X., Chen J., Gao F., Chen X., Masek J.G. (2010). An enhanced spatial and temporal adaptive reflectance fusion model for complex heterogeneous regions. Remote Sens. Environ..

[10] Zhu X., Helmer E.H., Gao F., Liu D., Chen J., Lefsky M.A. (2016). A flexible spatiotemporal method for fusing satellite images with different resolutions. Remote Sens. Environ..

[11] Song H., Liu Q., Wang Y., Hang G., Huang B. (2018). Spatiotemporal satellite image fusion using deep convolutional neural networks. IEEE J. Sel. Topics Appl. Earth Observ. Remote Sens..

[12] Tan Z., Yue P., Di L., Tang J. (2018). Deriving High Spatiotemporal Remote Sensing Images Using Deep Convolutional Network. Remote Sens..

[13] Tan Z., Di L., Zhang M., Guo L., Gao M., Yao Z. (2021). Generative adversarial networks for spatiotemporal data fusion. IEEE Trans. Geosci. Remote Sens..

[14] Chen G., Jiao P., Hu Q., Xiao L., Ye Z. (2022). SwinSTFM: Remote Sensing Spatiotemporal Fusion Using Swin Transformer. IEEE Trans. Geosci. Remote Sens..

[15] Liu X., Deng C., Chanussot J., Hong D. (2019). StfNet: A Two-Stream Convolutional Neural Network for Spatiotemporal Image Fusion. IEEE Trans. Geosci. Remote Sens..

[16] Lin L., Shen Y., Wu J., Nan F. (2023). CAFE: A Cross-Attention Based Adaptive Weighting Fusion Network for MODIS and Landsat Spatiotemporal Fusion. IEEE Geosci. Remote Sens. Lett..

[17] Huang H., He W., Zhang H. (2024). STFDiff: Remote sensing image spatiotemporal fusion with diffusion models. Inf. Fusion.

[18] Ma Y., Wang Q., Wei J. (2024). Spatiotemporal fusion via conditional diffusion model. IEEE Trans. Geosci. Remote Sens..

[19] Zhang Q., Zhang X., Quan C., Zhao T., Huo W., Huang Y. (2025). Mamba-STFM: A Mamba-based spatiotemporal fusion method for remote sensing images. Remote Sens..

[20] Guan B., Tan D., Tao J., Su A., Shang Y., Yu Q. (2026). Fusion-Restoration Image Processing Algorithm to Improve the High-Temperature Deformation Measurement. Exp. Mech..

[21] Wei H., Wang N., Liu Y., Ma P., Pang D., Sui X., Chen Q. (2024). Spatio-temporal feature fusion and guide aggregation network for remote sensing change detection. IEEE Trans. Geosci. Remote Sens..

[22] Li X., Peng Q., Zheng Y., Lin S., He B., Qiu Y., Chen J., Chen Y., Yuan W. (2024). Incorporating environmental variables into spatiotemporal fusion model to reconstruct high-quality vegetation index data. IEEE Trans. Geosci. Remote Sens..

[23] Ma Z., Bao W., Feng W., Zhang X., Ma X., Qu K. (2025). SFT-GAN: Sparse fast transformer fusion method based on GAN for remote sensing spatiotemporal fusion. Remote Sens..

[24] Li A., Xiao T. Spatio-temporal fusion assisted by gap filling under different cloud conditions. Proceedings of the Seventh International Conference on Geoscience and Remote Sensing Mapping (GRSM 2025).

[25] Chen J., Wang L., Feng R., Liu P., Han W., Chen X. (2021). CycleGAN-STF: Spatiotemporal Fusion via CycleGAN-Based Image Generation. IEEE Trans. Geosci. Remote Sens..

[26] Hu J., Shen L., Sun G. Squeeze-and-excitation networks. Proceedings of the IEEE Conference on Computer Vision and Pattern Recognition (CVPR).

[27] Li W., Cao D., Peng Y., Yang C. (2021). MSNet: A Multi-Stream Fusion Network for Remote Sensing Spatiotemporal Fusion Based on Transformer and Convolution. Remote Sens..

[28] Woo S., Park J., Lee J., Kweon I.S. CBAM: Convolutional block attention module. Proceedings of the European Conference on Computer Vision (ECCV).

[29] Liu Z., Lin Y., Cao Y., Hu H., Wei Y., Zhang Z., Lin S., Guo B. Swin transformer: Hierarchical vision transformer using shifted windows. Proceedings of the IEEE/CVF International Conference on Computer Vision.

[30] Ronneberger O., Fischer P., Brox T. (2015). U-net: Convolutional networks for biomedical image segmentation. International Conference on Medical Image Computing and Computer-Assisted Intervention.

[31] He K., Zhang X., Ren S., Sun J. Deep residual learning for image recognition. Proceedings of the IEEE Conference on Computer Vision and Pattern Recognition (CVPR).

[32] Emelyanova I.V., McVicar T.R., Van Niel T.G., Li L.T., Van Dijk A.I. (2013). Assessing the accuracy of blending Landsat–MODIS surface reflectances in two landscapes with contrasting spatial and temporal dynamics: A framework for algorithm selection. Remote Sens. Environ..

[33] Wald L., Ranchin T., Mangolini M. (1997). Fusion of satellite images of different spatial resolutions: Assessing the quality of resulting images. Photogramm. Eng. Remote Sens..

[34] Wang Z., Bovik A.C., Sheikh H.R., Simoncelli E.P. (2004). Image quality assessment: From error visibility to structural similarity. IEEE Trans. Image Process..

[35] Keshava N. (2004). Distance metrics and band selection in hyperspectral processing with applications to material identification and spectral libraries. IEEE Trans. Geosci. Remote Sens..

[36] Yuhas R.H., Goetz A.F.H., Boardman J.W. (1992). Discrimination among semi-arid landscape endmembers using the spectral angle mapper (SAM) algorithm. JPL, Summaries of the Third Annual JPL Airborne Geoscience Workshop. Volume 1: AVIRIS Workshop.

[37] Liu P., Wang L., Chen J., Cui Y. (2024). Semiblind Compressed Sensing: A Bidirectional-Driven Method for Spatiotemporal Fusion of Remote Sensing Images. IEEE J. Sel. Top. Appl. Earth Obs. Remote Sens..

